# Improvement of the aerodynamic behavior of a sport utility vehicle numerically by using some modifications and aerodynamic devices

**DOI:** 10.1038/s41598-022-24328-w

**Published:** 2022-11-24

**Authors:** Ahmed Al-Saadi, Khaled Al-Farhany, Kadhim K. Idan Al-Chlaihawi, Wasim Jamshed, Mohamed R. Eid, El Sayed M. Tag El Din, Zehba Raizah

**Affiliations:** 1 Department of Mechanical Engineering, University of Al-Qadisiyah, Al Diwaniyah, Al-Qadisiyah 58001 Iraq; 2 Department of Mathematics, Capital University of Science and Technology (CUST), Islamabad, 44000 Pakistan; 3 Department of Mathematics, Faculty of Science, New Valley University, Al-Kharga, 72511 Al-Wadi Al-Gadid Egypt; 4 Department of Mathematics, Faculty of Science, Northern Border University, Arar, 1321 Saudi Arabia; 5 Electrical Engineering, Faculty of Engineering and Technology, Future University in Egypt, New Cairo, 11835 Egypt; 6 Department of Mathematics, College of Science, King Khalid University, Abha, Saudi Arabia; 7 Abha, Saudi Arabia

**Keywords:** Mathematics and computing, Physics

## Abstract

The present study proposes aerodynamically optimized exterior designs of a sport utility vehicle using computational fluid dynamics analysis based on steady-state Reynolds-averaged Navier–Stokes turbulence models. To achieve an optimal design, modifications of the outer shape and adding some aerodynamic devices are investigated. This study focuses on modifying this vehicle model’s upper and front parts. At the same time, the rear diffuser and spare tire on the back door as a fairing are used as aerodynamic devices for improving streamlines. All these modifications and add-on devices are simulated individually or in combination to achieve the best exterior design. A variety of Reynolds numbers are used for determining the optimization variables. Tetrahedral cells are used throughout the global domain because of the sharp edges in the geometry of the Discovery car model. At the same time, prism cells around car surfaces are adopted to improve the accuracy of the results. A good agreement between the numerical drag coefficient in the present study for the baseline models and the experimental data has been achieved. Changes in the drag and lift coefficients are calculated for all models. It is clear from the numerical results that the use of combined modifications and add-on devices has a significant effect in improving the overall aerodynamic behavior. As a result, the drag coefficient for the optimal design of the Discovery 4th generation is reduced from 0.4 to 0.352 by about 12% compared to the benchmark. Simultaneously, the lift coefficient is 0.037 for optimal design, and it is an acceptable value. It is found that combining all optimal modified configurations can improve both *C*_*D*_ and *C*_*L*_ simultaneously.

## Introduction

Most of the braking system research focuses on advancing current technologies, such as active suspension systems, automatic anti-lock braking, traction control devices, and brake disk thermal characteristics. Researchers are also being carried out in the field of technologies that assist the driver by providing a warning or automatically commencing brakes when a collision risk is recognized. Despite its relevance, only a little research on how aerodynamics might improve the braking characteristics of passenger vehicles and its possibilities can be discovered in the literature. Most research yet has focused on high-performance vehicles like racing cars and high-end sports cars, making it difficult to determine how far this innovation can be transferred to passenger automobiles. It should be remembered that active aerodynamics was outlawed from racing in the late 1960s. One of the reasons for this choice was a safety concern, as moveable parts were not as firmly mounted as the problems were affecting. Active aerodynamic components were utilized in racing automobiles to boost performance, which was sometimes more vital than the driver's safety. The active aerodynamic devices that were first utilized in racing vehicles were created with limited knowledge of how they worked. As a result, they were more likely to have catastrophic defects that revealed themselves when the vehicles performed to their maximum ability during a race. Aerodynamic forces were frequently passed straight to the unsprung sections of the suspension, which conveyed road shocks to the wing's nodes. When looking at photographs of cars from that era, one can see that the designers understated the increase in a vehicle's performance when driving around a bend, leading to greater lateral pressure conveyed by the tires also and acting on the highly placed wings, which could outcome in the wing dissociating from the car. The only exception to the energetic aerodynamics bans in Formula One today is a drag reduction framework, which consists of a movable airfoil that lowers its angle of attack on straightaways to generate flow separation on the main airfoil, reducing downward force and the high parasitic drag produced on the very low aspect wing and minimizing total drag. The usage of rear wings and spoilers in braking mode is frequent in supercars, demonstrating that active aerodynamic features are protected in operation if properly built. The exterior design of road vehicles has a great influence on fuel consumption. Streamlines vehicle shapes can reduce fuel consumption, which reduces pollution^1^. Using smooth curved surfaces and aerodynamic devices helps achieve streamlined geometries^2–4^. The aerodynamic behavior of automobiles can be tested experimentally by using a wind tunnel or numerically by using CFD^5^.

Relatively sharp edges and semi-straight lines characterize the exterior design of the Land Rover Discovery 3rd generation. A stepped roofline is the main characteristic of this vehicle. Then came the 4th generation of this vehicle with slight improvements to the exterior design. A wind tunnel with a fixed ground supported by a limited numerical approach was used to develop this SUV^6,7^. That leads to a reduced drag coefficient from 0.41 to 0.4^8–10^. In 2017, the 5th generation of Land Rover Discovery appeared with a new streamlined exterior design. This model was investigated experimentally and numerically to calculate the drag coefficient^11^. Different aerodynamic devices were tested on the Land Rover Discovery 4th generation (LRD4) to achieve optimal aerodynamic behavior^12,13^.

SUVs and station wagon cars have more air resistance than sedan models. Due to a high rake angle, the airflow separates early in such vehicles at the roof end. This phenomenon can lead to a rise the drag resistance due to a high degree of turbulence behind the vehicle^14,15^. The rear screen and rear fairing are examples of passive aerodynamic devices. These add-on devices were investigated experimentally with a scale SUV model to reduce ${C}_{D}$^16^. In comparison, base bleed and rear cavities were analyzed experimentally for a full-scale SUV model to achieve the greatest drag reduction^17^. Closed and open cooling apertures for SUVs were studied experimentally to know their effect on drag resistance^18^. Pressure and drag coefficients were examined experimentally and numerically for the 1/5th Fiat Linea model in different blockage ratio magnitudes to investigate the effect of the blockage ratio on the ${C}_{D}$^19^. A rear diffuser was studied for a sedan car using a numerical method to decrease ${C}_{D}$ by about 4%^20^. The base pressure is most important to calculate in the aerodynamics of SUVs because it can result in about half of the overall air resistance^21^. Some add-on devices are suitable for improving the lift coefficient but negatively affect the drag coefficient. A rear spoiler similar to a wing was used to increase the car's stability on the road and reduce noise^22^. The optimizing exterior design of a passenger car can minimize air resistance. As proof, the $C_{D}$ was reduced by modifying the rear part exterior design of the Sonata model^23^. $k - \varepsilon$ turbulence model can be adopted to achieve accurate simulation results for the aerodynamics of passenger cars^24–27^.

Many studies have focused on the reduction of $C_{D}$ in ground vehicles. Therefore, the major aim of the present study is to reduce $C_{D}$ and improve the stability of LRD4 on the road without affecting the capacity, comfort, and the main dimensions of this vehicle model. A range between 7.3 × 10^6^ and 14.6 × 10^6^ of $Re$ is used to investigate its effect on the $C_{D}$ and $C_{L}$. Many suggested modifications and aerodynamic devices are investigated to achieve the primary goal. Combining improvements and add-on devices is used in the current study to reach the best model. Creating a balance between $C_{D}$ and $C_{L}$ is adopted in the current study to achieve the optimal design.

## Governing equations and numerical method

SolidWorks (version 2014) software is used to create all vehicle geometries in the present study, while all numerical simulations are achieved by using ANSYS Fluent^28–30^.

### Governing equations

The motion of fluid flow can be represented through the governing equations. Navier Stokes equations, governing equations, are based on the conservation of momentum, mass, and energy. Most numerical studies of external flow are based on RANS equations^31^. All simulations are achieved by using a realizable *k–ε* turbulence model. The following transport equations are used for $k$ and $\varepsilon$^32^

1 $$\frac{{\partial \left( {\rho k} \right)}}{\partial t} + div\left( {\rho k{\varvec{U}}} \right) = div\left[ {\frac{{\mu_{t} }}{{\sigma_{k} }}grad{ }\left( k \right)} \right] + 2\mu_{t} S_{ij} \cdot S_{ij} - \rho \varepsilon ,$$

2 $$\frac{{\partial \left( {\rho \varepsilon } \right)}}{\partial t} + div\left( {\rho \varepsilon {\varvec{U}}} \right) = div\left[ {\frac{{\mu_{t} }}{{\sigma_{\varepsilon } }}grad \left( \varepsilon \right)} \right] + C_{1\varepsilon } \frac{\varepsilon }{k}2\mu_{t} S_{ij} \cdot S_{ij} - C_{2\varepsilon } \rho \frac{{\varepsilon^{2} }}{k}.$$

The following equation is the eddy viscosity^32^

3 $$\mu_{t} = \rho C_{\mu } \frac{{k^{2} }}{\varepsilon }.$$

The following equations are the transport equations in the realizable $k - \varepsilon$ model for $k$ and $\varepsilon$

4 $$\frac{\partial }{\partial t}\left( {\rho k} \right) + \frac{\partial }{{\partial x_{j} }}\left( {\rho k{\varvec{u}}_{{\varvec{j}}} } \right) = \frac{\partial }{{\partial x_{j} }}\left[ {\left( {\mu + \frac{{\mu_{t} }}{{\sigma_{k} }}} \right)\frac{\partial k}{{\partial x_{j} }}} \right] + G_{k} + G_{b} - \rho \varepsilon - Y_{M} + S_{k} ,$$

5 $$\frac{\partial }{\partial t}\left( {\rho \varepsilon } \right) + \frac{\partial }{{\partial x_{j} }}\left( {\rho \varepsilon {\varvec{u}}_{{\varvec{j}}} } \right) = \frac{\partial }{{\partial x_{j} }}\left[ {\left( {\mu + \frac{{\mu_{t} }}{{\sigma_{\varepsilon } }}} \right)\frac{\partial \varepsilon }{{\partial x_{j} }}} \right] + \rho C_{1} S_{\varepsilon } - \rho C_{2} \frac{{\varepsilon^{2} }}{{k + \sqrt {v\varepsilon } }} + C_{1\varepsilon } \frac{\varepsilon }{k}C_{3\varepsilon } G_{b} + S_{\varepsilon } .$$

The $C_{D}$ is calculated based on the following equation^5^

6 $$C_{D} = \frac{{2{ }F_{D} }}{{\left( {\rho { }V^{2} { }A} \right)}}.$$

While the following equation is used to calculate $C_{L}$^5^

7 $$C_{L} = \frac{{2{ }F_{L} }}{{\left( {\rho { }V^{2} { }A} \right)}}.$$

### Vehicle model

**Figure 1:**
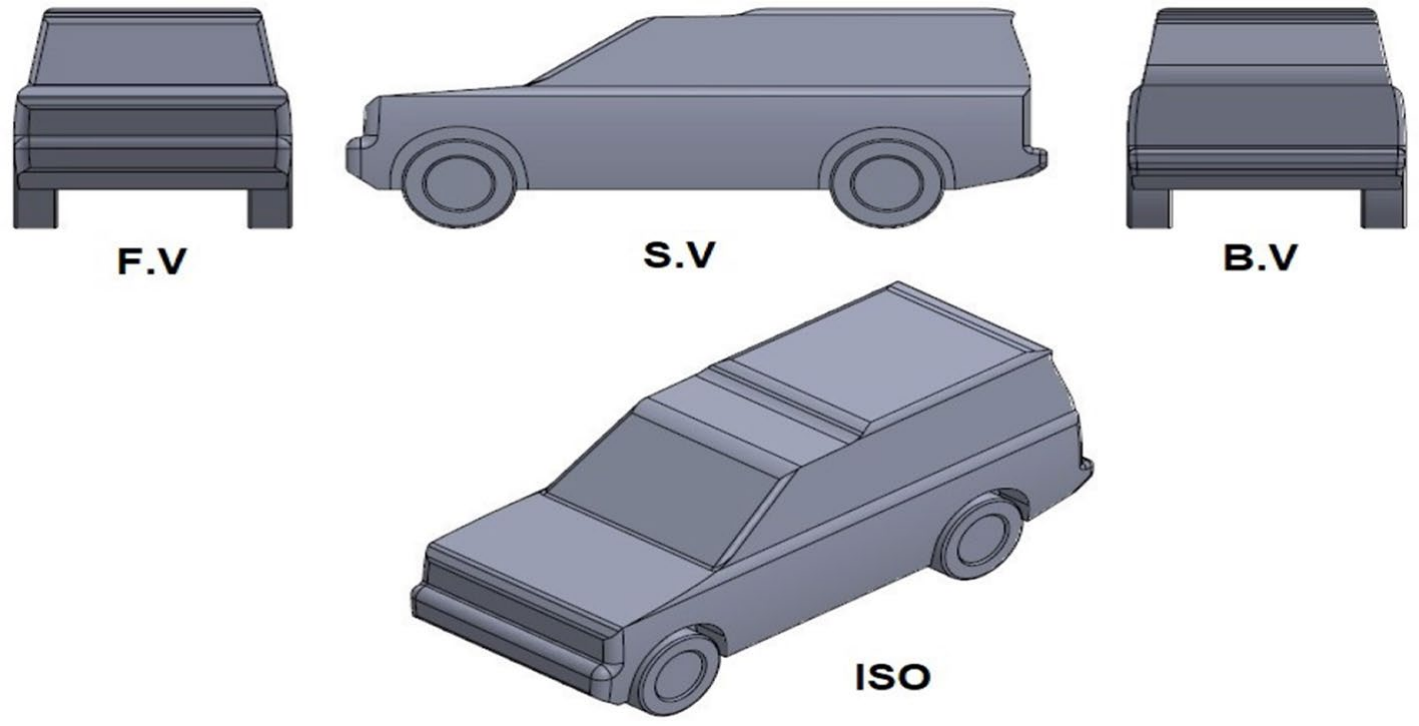
The benchmark of the LRD4.

SUVs have been more prevalent in the last decades because they are safer than other models and have more cargo room. Therefore, studying and improving SUVs are more important than other vehicle models. LRD4 is used in the present study as a benchmark for SUVs because numerous academic references describe the car's properties and overall aerodynamic behavior^33–37^. This model has a 0.4 drag coefficient because of its non-streamlines exterior design^8–11^. SolidWorks software is used to create this computational model. Overall length, height, width without side mirrors, and the wheelbase of the LRD4 are 4.835 m, 1.887 m, 1.915 m, and 2.51 m, respectively^9^. Figure 1 shows the main views of the 3-D full-size benchmark of the LRD4.

### Computational domain

A cube tunnel is used as a computational domain in the current study. Two different sizes of the computational domain are used. The first size is similar to the wind tunnel used in experimental tests to validate the numerical simulation results. While extra dimensions are used in the second computational domain, as recommended by Al-Saadi^38^. The width and height of the second computational domain are set larger than in a MIRA wind tunnel^39^ to avoid the effect of a possible wall boundary layer. A frontal projected area of this computational domain is 123.66 m^2^.

**Figure 2:**
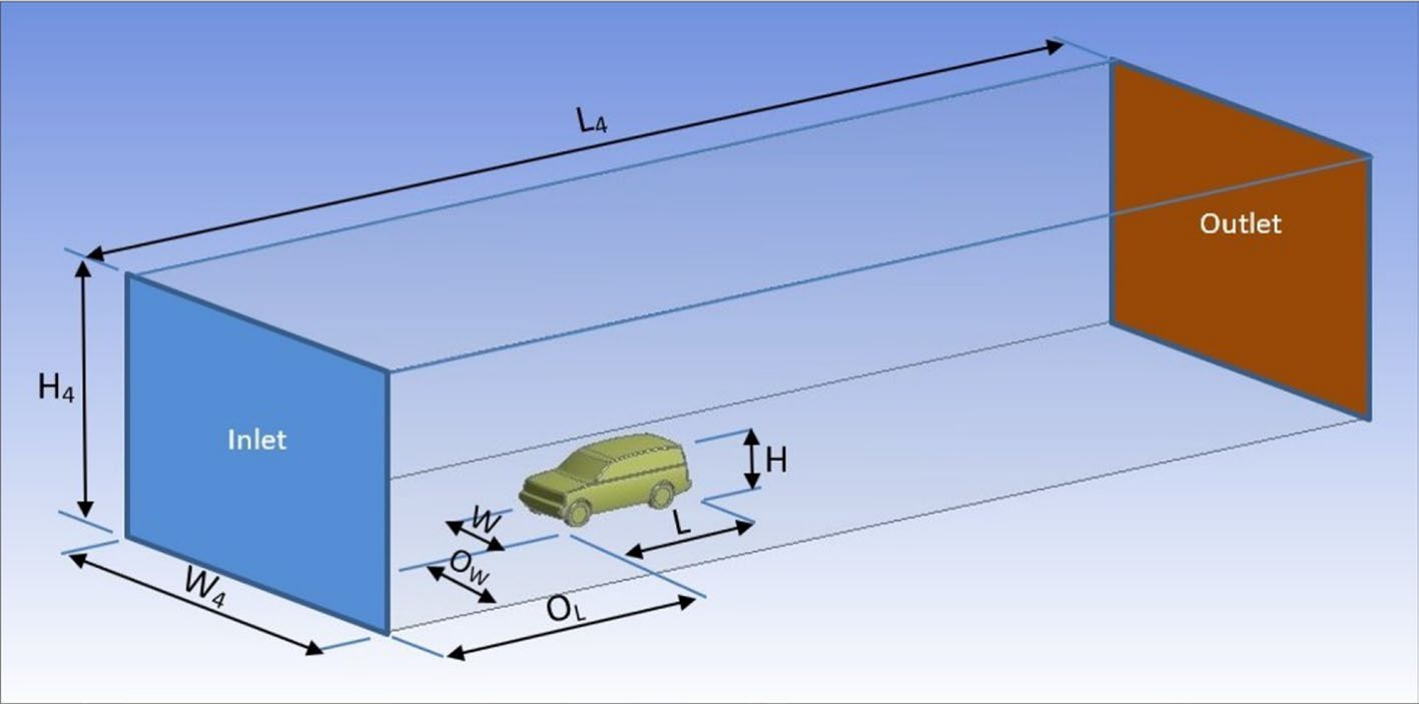
Computational domain for numerical simulations.

The computational domain length is about 8.5 of the LRD4 length. Figure 2 illiterates the whole geometry of the LRD4 inside the computational domain. Dimensions of the computational domain, vehicle geometry, and some parameters are presented in Table 1. The global domain can be regarded as an asymmetric system. Therefore, half of the vehicle geometry and the computational domain is used in all simulations to reduce the simulation time.

**Table 1:** Dimensions of the whole system and some parameters.

Dimensions of the computational domain	${{H}}_{{{4}}}$	8.887 m
	$W_{4}$	13.915 m
	$L_{4}$	41.1 m
Parameters of the computational domain	$O_{H}$	7 m
	$O_{W}$	6 m
	$O_{L}$	15 m
Dimensions of the LRD4 model	$H$	1.887 m
	$W$	1.915 m
	$L$	4.835 m
Mass flow rate	$m^{o}$	2103.923 kg/s
Blockage ratio	$B$	2.435%

### Numerical grids

**Figure 3:**
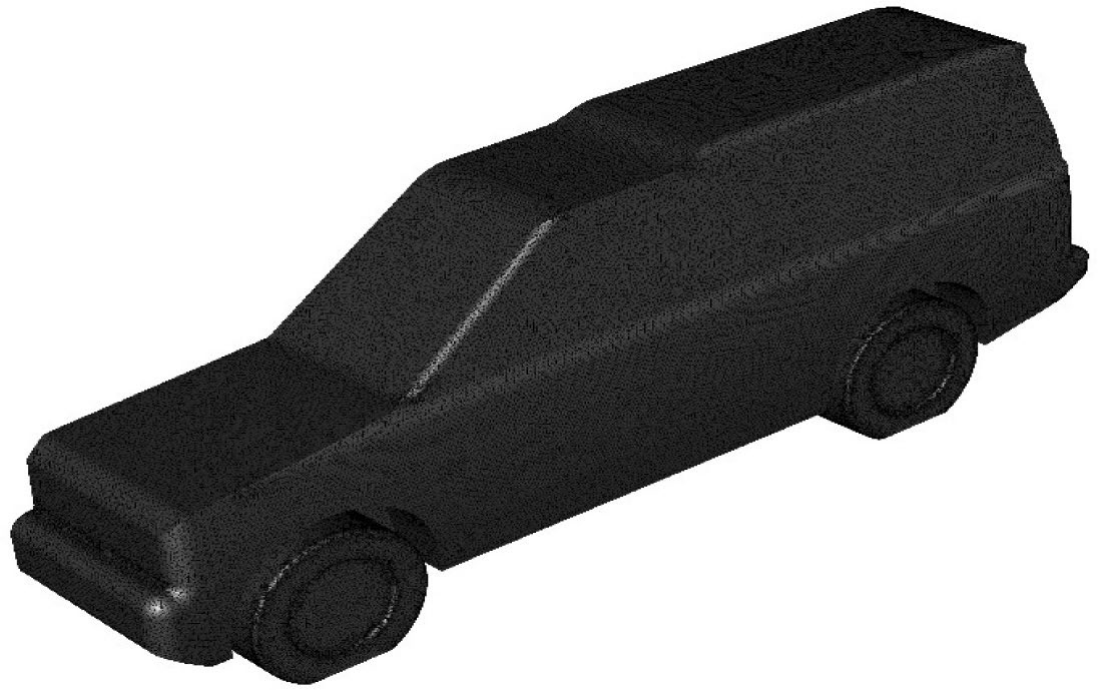
Surface mesh of the LRD4.

**Figure 4:**
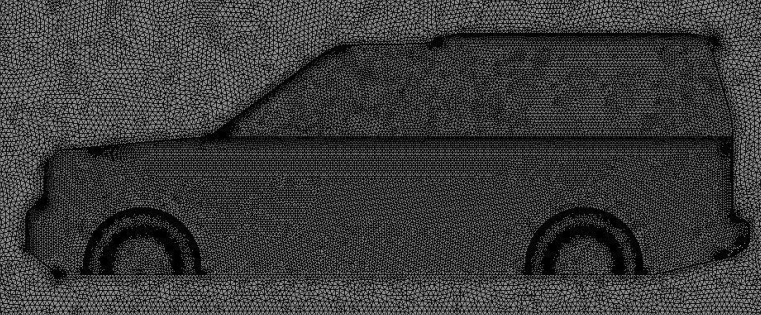
Tetrahedral mesh with 5 prism layers over the surfaces of the LRD4.

Mesh quality in the whole computational domain is an extremely critical step in numerical simulation. The unstructured tetrahedral cells in the global domain are generated by using ANSYS meshing with varying levels of refinement. This type of mesh is used to cope with the complexity of vehicle geometry. Figure 3 shows half LRD4 model surface mesh. The objective is to predict the air properties close to the car's surfaces precisely, 5 prismatic cell layers are used around the vehicle's exterior surfaces and over the road. Three VCRs are used around the vehicle's geometry to achieve more control on growth ratio and mesh density. About 13 × 10^6^ cells for half computational domain are chosen for all simulations after some testing for computational time and accuracy. The optimal mesh for the benchmark of the LRD4 model is shown in Fig. 4.

Depending on where it concerned the vehicle's surface, y+ employed an aspect ratio smaller than 30 for all Discovery car configurations in the current investigation. For this reason, the realizable *k–ε* model was more accurate than other turbulence models^32,37^.

### Boundary conditions

The following conditions are applied for simulations in the current study: uniform inlet velocities from the frontal side of the computational domain with a wide range of Reynolds numbers (*Re* between 7.3 × 10^6^ and 14.6 × 10^6^). The current study applies the mass flow rates (${\dot{\text{m}}}$) between 3687.186 kg/s and 6816.9 kg/s. All properties of air are calculated at 15 °C. A stationary wall with no slip is used as a boundary condition for the sidewalls and top wall of the computational domain. In numerical simulations, all vehicle tires are unmoving similar to the experimental model. The underbody surface of the vehicle is flat in this numerical study to simplify the mesh.

### Numerical set-up

Realizable *k–ε*, standard *k–ω,* and SST *k–ω* turbulence models were applied in the current study. If the Reynolds number is low, the *k–ω* turbulence model is accurate near the wall surface^32,36^. For high Reynolds number flows, near the wall surface does not require resolution due to the wall function option in the *k–ε* turbulence model. This technique significantly reduces computational time^37^. A realizable *k–ε* model has been extensively adopted for simulations of the external aerodynamics of cars as it provides good results in an equitable amount of computational time^24^. The second-order upwind scheme was used for the momentum, turbulent kinetic energy, and turbulent dissipation rate. The second-order choice was used for the pressure because it can provide more accurate numerical results than the first-order in the case of using tetrahedral meshes. In the set-up, 0.25 was used for the relaxation factor.

## Results and discussion

### Validation of CFD analysis

$C_{D}$ of LRD4 is measured experimentally in the MIRA full-scale wind tunnel^9,10,38^. The tunnel test section has a cross-sectional area of 34.9 m^2^, while the overall length of the MIRA tunnel is only 15 m^38^. All experimental tests were achieved at a velocity of 100 km/h and zero yaw angle. The computational domain with a similar size to MIRA wind tunnel is investigated in the current study to examine the numerical simulation results. Numerical simulation of the benchmark of the LRD4 is done using the same boundary conditions of the experimental work^38^.

**Figure 5:**
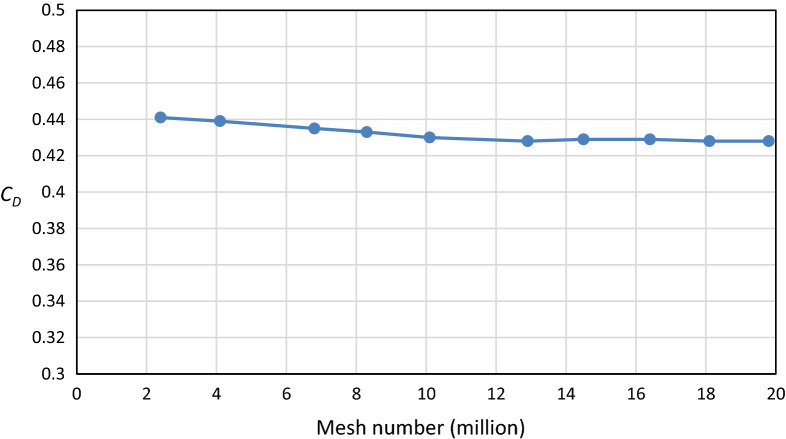
Grid dependency test.

A critical analysis of the number of cells was performed to reach the optimal number of mesh. Ten simulations for the benchmark were performed with different mesh resolutions and examined to see if the numerical results changed. The first simulation was done using a coarse mesh for the baseline model. Then, gradually refining the mesh for the next simulations was accomplished. This process continued until the changes observed in the simulation results were smaller than an acceptable error. The percentage error of the drag coefficient in the simulation result for the first mesh resolution was 10.25 when using the coarse mesh, which has about 2.4 million cells. Then, this percentage error decreased until it reached 7% at about 26 million cells. The analysis of mesh independence in the current study for benchmarking shows that numerical results change with further mesh refinement. A balance between the accuracy of numerical simulation results and the computation time are taken into consideration. Figure 5 depicts a grid dependency analysis using the realizable *k–ε* model with a computational domain size similar to the MIRA wind tunnel. The drag coefficient is affected by the number of cells, up to about 26 million cells for the whole computational domain, beyond which no consequential mesh dependency is noticeable.

Through convergence equations for the *k–ε* model (*k*, epsilon, continuity, and velocities in three dimensions), it is clear that after about 800 iterations, the solution of these equations no longer changes. The residuals of *k* and epsilon were less than 5 × 10^–4^. Also, the residuals of all velocities are reduced to less than 10^–6^. Only the residual of continuity decreased to less than 5 × 10^–3^. Each case of simulation run needs at least 36 h to finish.

Table 2 shows the $C_{D}$ and $C_{L}$ obtained from numerical simulation of the benchmark model of the LRD4 are compared with the experimental data^9,10^. The numerical simulations provided great agreement with all experimental data.

**Table 2:** $C_{D}$ and $C_{L}$ of the benchmark of the LRD4 for experimental and numerical results.

Parameter	Numerical results	Experimental data
$H_{4}$ (m)	4.4	4.4
$L_{4}$ (m)	15	15
$W_{4}$ (m)	7.9	7.9
$C_{D}$	0.428	0.4
$C_{LF}$ (lift coefficient on the front wheels)	0.05	0.06
$C_{LR}$ (lift coefficient on the rear wheels)	− 0.035	− 0.03
Blockage ratio ($\%$)	9.025	9.025
Percentage error of $C_{D}$ ($\%$)	7	–

### Modified models

Most vehicle engine power is used to overcome the air resistance at high speed, especially with a non-streamlined vehicle design. The benchmark of the LRD4 has a high $C_{D}$. Therefore, redesigning the exterior shape and placing some add-on devices on the exterior surfaces of the LRD4 can reduce $C_{D}$. Redesigning the LRD4 by modifying the upper and front regions is represented the first modified model. All these modifications are used to create a more streamlined geometry.

**Figure 6:**
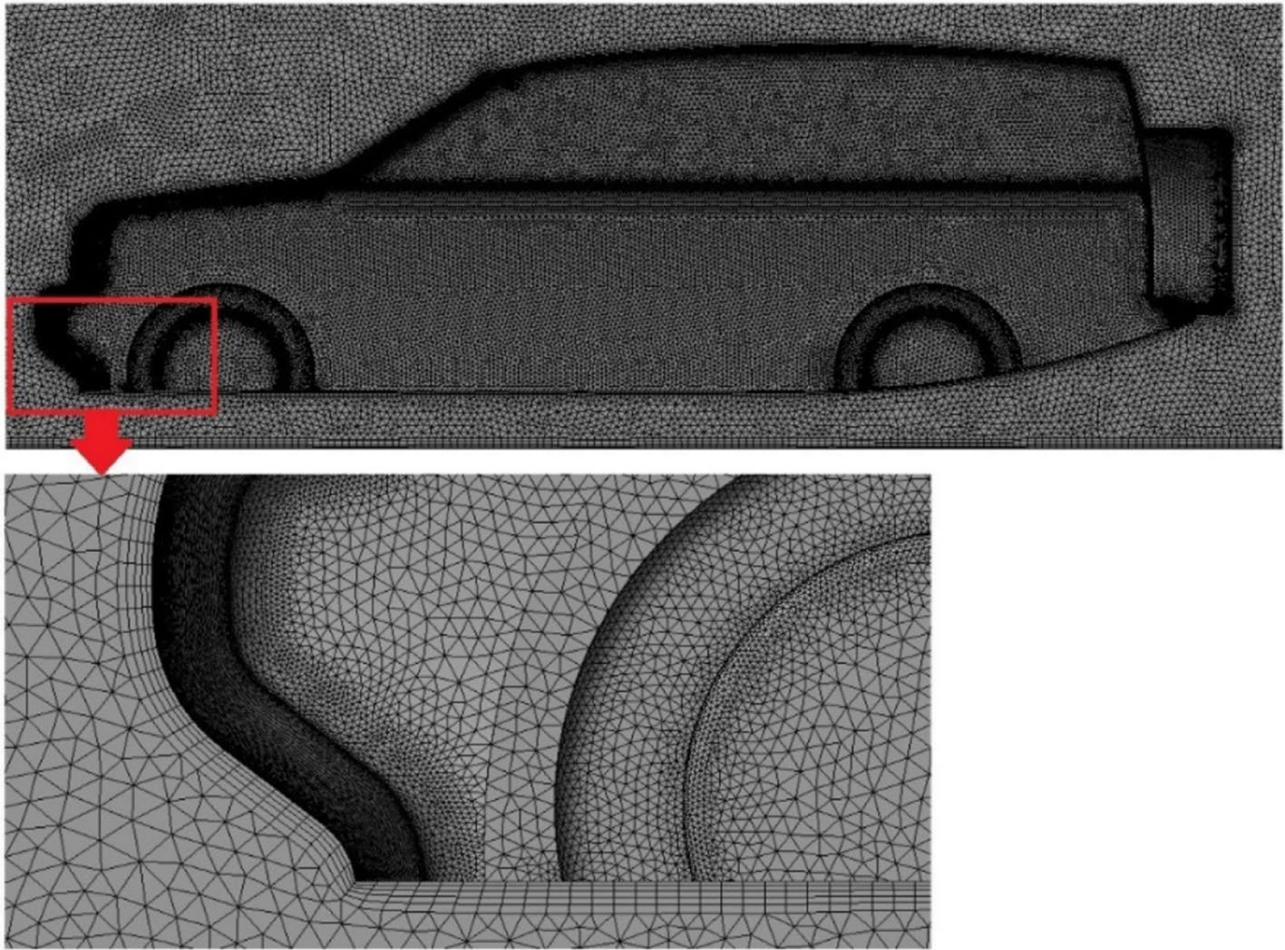
Mesh of the modified LRD4 with a close-up at the front bumper.

Placing a spare tire behind the vehicle as fairing is the second modified model for the LRD4. Another modified model is created by adding a diffuser to the modified LRD4. The last modified model is performed by combining all modifications of the LRD4. The computational domain of redesigned LRD4 and LRD4 with aerodynamic device models is similar to the computational domain of the benchmark of the LRD4. Unstructured tetrahedral mesh with three VCRs is used for all modified LRD4 models throughout the computational domain. Figure 6 shows five prismatic cells over the surfaces of all modified LRD4 models in order to provide accurate numerical results.

#### Modified exterior design model

**Figure 7:**
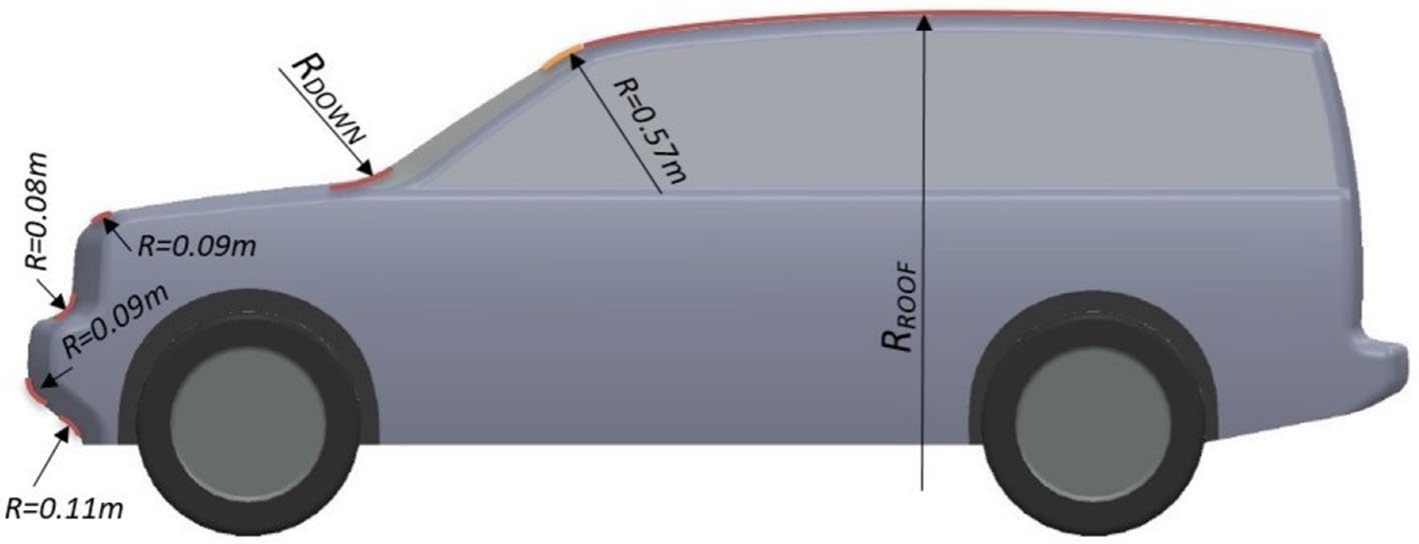
Side view of modified LRD4.

Some modifications for the exterior design of LRD4 are proposed while preserving the main car dimensions except the overall height. The main modification is in the upper part of the LRD4 to achieve a curved roof. Many different sizes are tested in order to achieve the optimal roof dimensions for LRD4. Some sharp edges in the front part of the LRD4 are changed to soft curved. Figure 7 shows all proposed modifications to LRD4, while Table 3 illustrates all proposed dimensions.

**Table 3:** $C_{D}$ and $C_{L}$ for many configurations of the exterior modifications of LRD4 model.

Config	$R_{DOWN}$ (m)	$R_{ROOF}$ (m)	$C_{D}$	$C_{L}$
1	0.3	8	0.381	0.147
2	0.4	8	0.379	0.142
3	0.5	8	0.378	0.139
4	0.3	9	0.376	0.137
5	0.4	9	0.372	0.132
6	0.5	9	0.371	0.13
7	0.3	10	0.369	0.129
8	0.4	10	0.365	0.126
9	0.5	10	0.361	0.125
10	0.3	11	0.371	0.127
11	0.4	11	0.366	0.125
12	0.5	11	0.362	0.124

All configurations of the modified exterior design of the LRD4 have $C_{D}$ less than the benchmark model and acceptable $C_{L}$ but higher than the benchmark model. This is because the modified model has a more streamlined shape than the benchmark model, which means fewer vortices. Configuration 9 in Table 3 has the minimum $C_{D}$ (0.361) and the most acceptable $C_{L}$ (0.125). As a result, the optimal design of these modifications (configuration 9) improved $C_{D}$ by about 9.75% compared with the benchmark.

#### Adding aerodynamic devices

**Figure 8:**
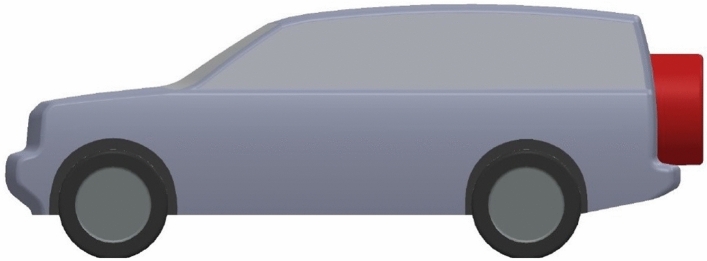
Side view of modified LRD4 with the spare tire.

**Figure 9:**
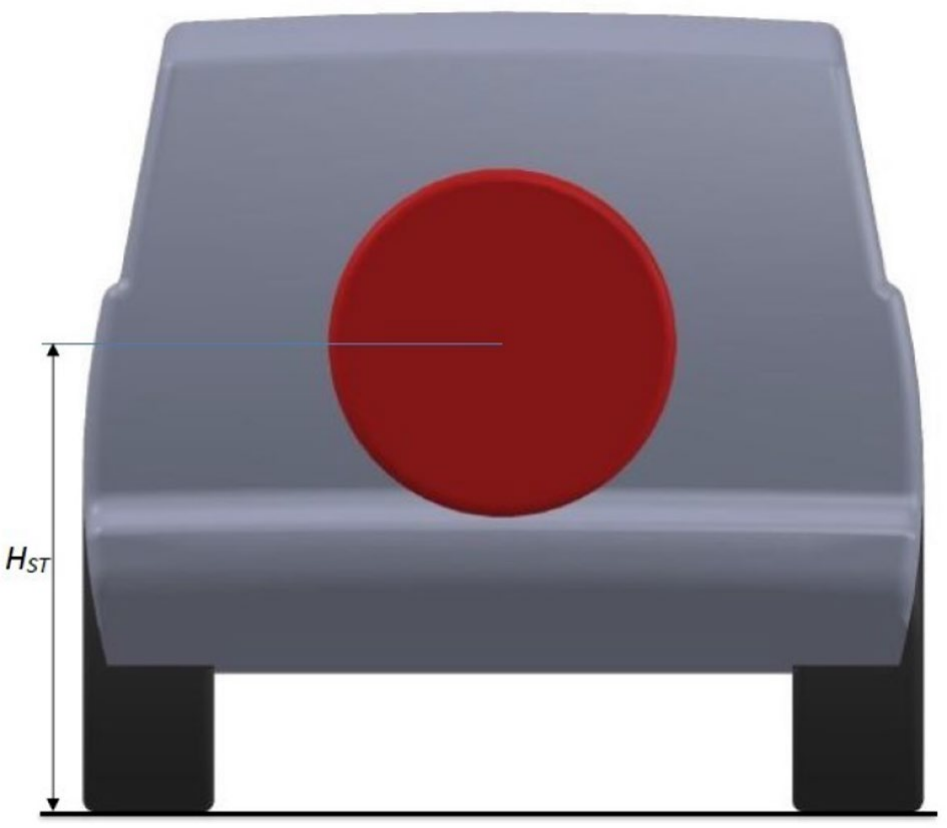
Back view of modified LRD4 with the spare tire.

In addition to exterior design modifications, two aerodynamic devices are investigated for the modified LRD4 model. Use a spare tire on the back door as a fairing to achieve a more streamlined model. The Discovery car with a spare tire on the back door has a better-streamlined look than the benchmark and modified models because the spare tire covers a part of the wake zone behind this car. This model has a lower pressure zone behind it. Figures 8 and 9 show the spare tire on the back door of the modified LRD4 model. The spare tire is placed in width in the middle of the back door, but many vertical positions are tested, as shown in Table 4, to achieve the optimal position. All cases of the spare tire position produced $C_{D}$ Lower than benchmark and redesign models, but these cases have $C_{L}$ between the benchmark and redesign models. In general, all configurations of the spare tire model have downforce more than the redesigned models. The optimal position of the spare tire is accomplished by configuration 4, as shown in Table 4. A reduction of about 10.75% is achieved by adding a spare tire to the modified LRD4.

**Table 4:** Multi configurations of the modified LRD4 with the spare tire.

Config	$H_{ST}$ (m)	$C_{D}$	$C_{L}$
1	1.07	0.357	0.039
2	1.08	0.357	0.039
3	1.09	0.357	0.039
4	1.1	0.357	0.038
5	1.11	0.358	0.038
6	1.12	0.358	0.037
7	1.13	0.358	0.037
8	1.14	0.358	0.036
9	1.15	0.358	0.036

**Figure 10:**
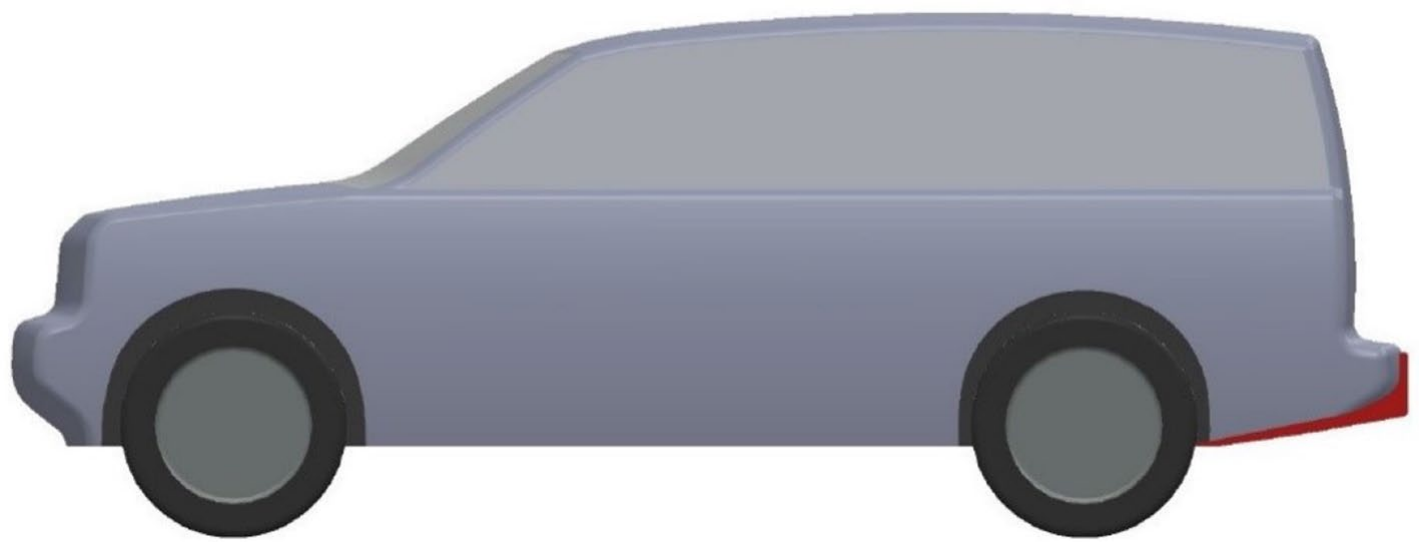
Side view of modified LRD4 with the spare tire.

**Figure 11:**
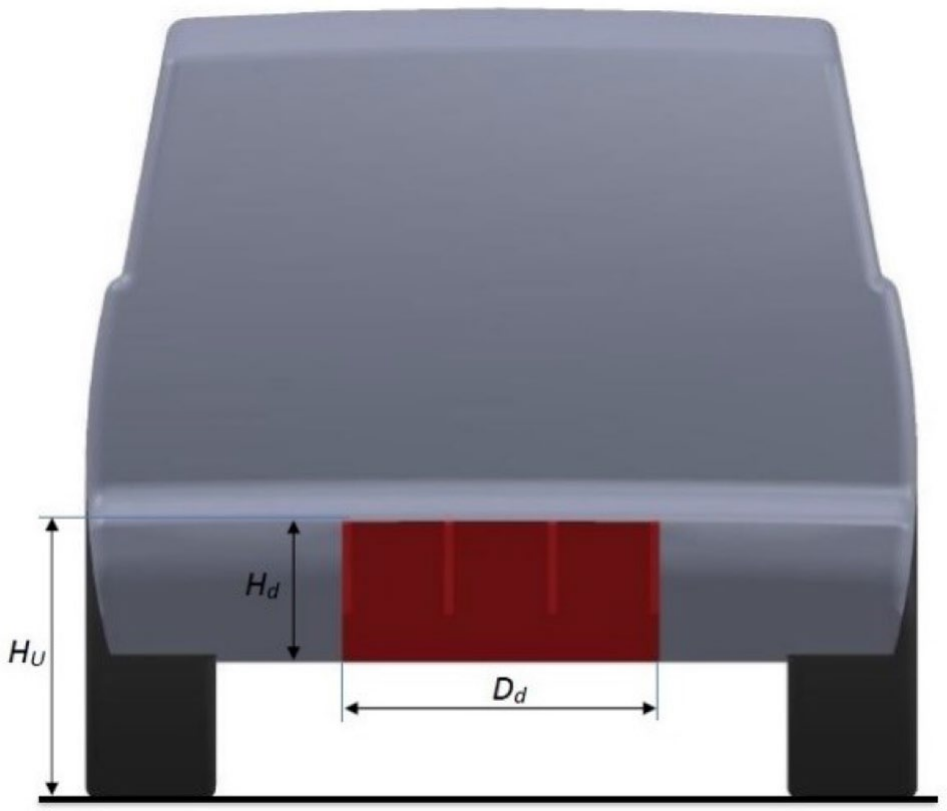
Back view of modified LRD4 with the spare tire.

Using a diffuser under the rear bumper is the second proposed aerodynamic device for the modified LRD4, as shown in Figs. 10 and 11. Many different dimensions of the diffuser are suggested, as shown in Table 5. Other dimensions of the diffuser under the rear bumper of the LRD4 are investigated to achieve the optimal design. A diffuser works to guide airflow into the core of a wake. This technique leads to fewer vortices in low-pressure areas. The best drag coefficient and downforce are achieved using the dimensions in configuration 8, as shown in Table 5. This add-on device has reduced the $C_{D}$ by 10.25%.

**Table 5:** Many configurations of the LRD4 with a diffuser.

Config	$H_{d}$ (m)	$D_{d}$ (m)	$C_{D}$	$C_{L}$
1	0.3	0.8	0.366	0.103
2	0.3	0.9	0.365	0.101
3	0.3	1	0.363	0.099
4	0.32	0.8	0.363	0.096
5	0.32	0.9	0.361	0.094
6	0.32	1	0.361	0.094
7	0.33	0.8	0.361	0.089
8	0.33	0.9	0.359	0.086
9	0.33	1	0.36	0.088
10	0.34	0.8	0.362	0.093
11	0.34	0.9	0.36	0.092
12	0.34	1	0.361	0.093

#### Combined modifications and add-on devices

**Figure 12:**
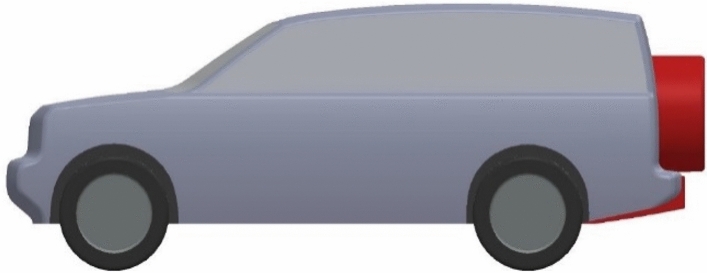
Back view of modified LRD4 with the spare tire.

Combined optimal modifications and aerodynamic devices can reduce the drag coefficient, which leads to reduced fuel consumption. Figure 12 shows LRD4 after using all optimal modifications and add-on devices. The minimum drag coefficient ($C_{D} = 0.352$) is achieved by combining redesign, spare tire, and diffuser configurations. These aerodynamic techniques reduced the drag coefficient by about 12%. The $C_{L}$ of the combined model is 0.037, and it is an acceptable value.

### Optimal design for the LRD4

**Figure 13:**
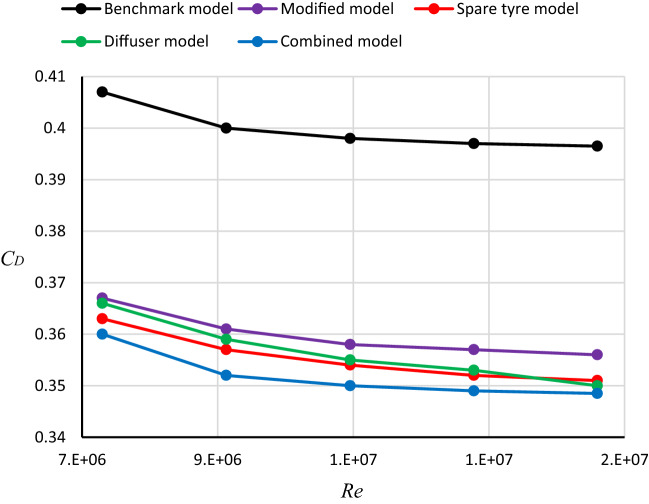
Relation between *C*_*D*_ and *Re* for many configurations of the LRD4.

Four models of the modified exterior design of the LRD4 (redesign, spare tire, diffuser, and combined model) are analyzed to achieve an optimal LRD4 model. Figure 13 shows the relation between $C_{D}$ and $Re$ for the benchmark and four different modified configurations of the LRD4 by using a computational domain as recommended by Al-Saadi^38^. It is clear that increasing in $Re$ leads to a decrease in $C_{D}$ as shown in all cases in Fig. 13. The best-modified model for this type of SUVs is combined because it has the minimum $C_{D}$ for a wide range of $Re$. The $C_{D}$ of the benchmark model is 0.4, while 0.352 for the combined modified model of the LRD4. $C_{D}$ of the optimal design is less than $C_{D}$ of the benchmark by about 12%. As a result of all numerical investigations in the current study, combined optimal modifications and aerodynamic devices can reduce the drag coefficient, which leads to reduced fuel consumption.

#### Velocity profile

**Figure 14:**
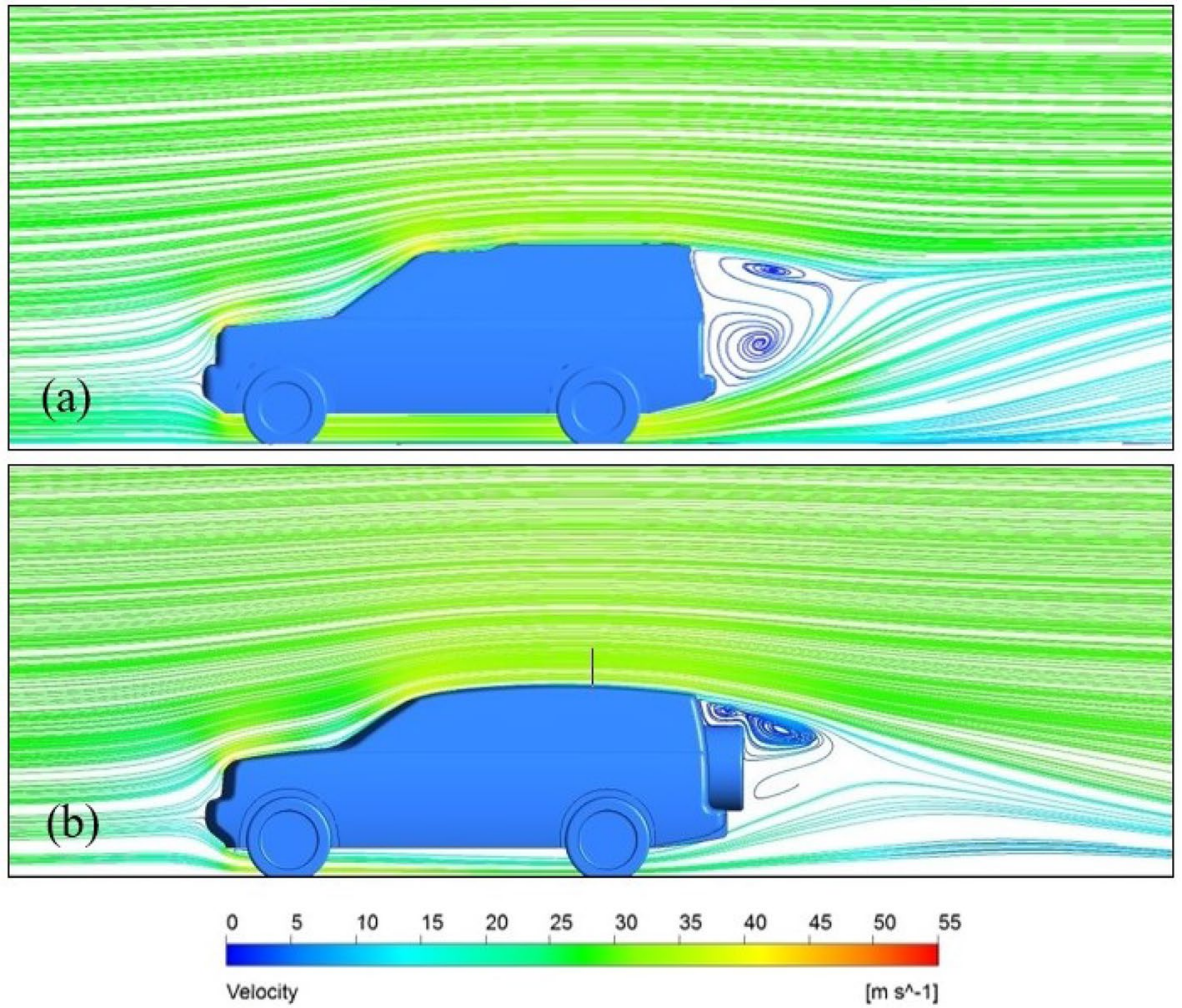
Streamlines on the symmetry plane for two models of the LRD4: (**a**) the benchmark and (**b**) the modified model with diffuser and spare tire.

Figure 14 shows the velocity profile around the benchmark and combined modified LRD4 model. The initial air velocity in these numerical simulations is similar to the experimental investigation (28 m/s). The airflow over the roof and underbody of the vehicle leads to generating two vortices behind the vehicle. The low pressure behind the benchmark of the LRD4 is the main reason for making vortices as non-uniform flow close to the rear bumper and roof end edge. While in the modified LRD4 model, these vortices are lower than the benchmark model because the spare tire covers some wake zone, and the diffuser underbody is directed air toward the core of the wake. The range of air velocity around the benchmark model is between zero and 45 m/s, as shown in Fig. 14. The maximum air velocity is at the leading edge of the benchmark of the LRD4 because of the reduction in the computational domain cross-sectional area. At the same time, the maximum air velocity for the modified model is less than the benchmark model due to reducing the overall height of the modified model. The air velocity decreases with increasing the cross-sectional area of the computational domain while maintaining a constant flow rate. By adding a diffuser to the rear underbody of the LRD4, swirling flow near the rear bumper is significantly reduced due to the air being directed to the wake zone. Vortices behind the LRD4 reduce by placing the spare tire on the back door of this model, but some vortices in the top of the spare tire zone slightly increased. Redesigning the LRD4 with a streamlined exterior shape and adding a diffuser and spare tire resulted in more airflow streamlines than the benchmark model.

#### Pressure distributions

**Figure 15:**
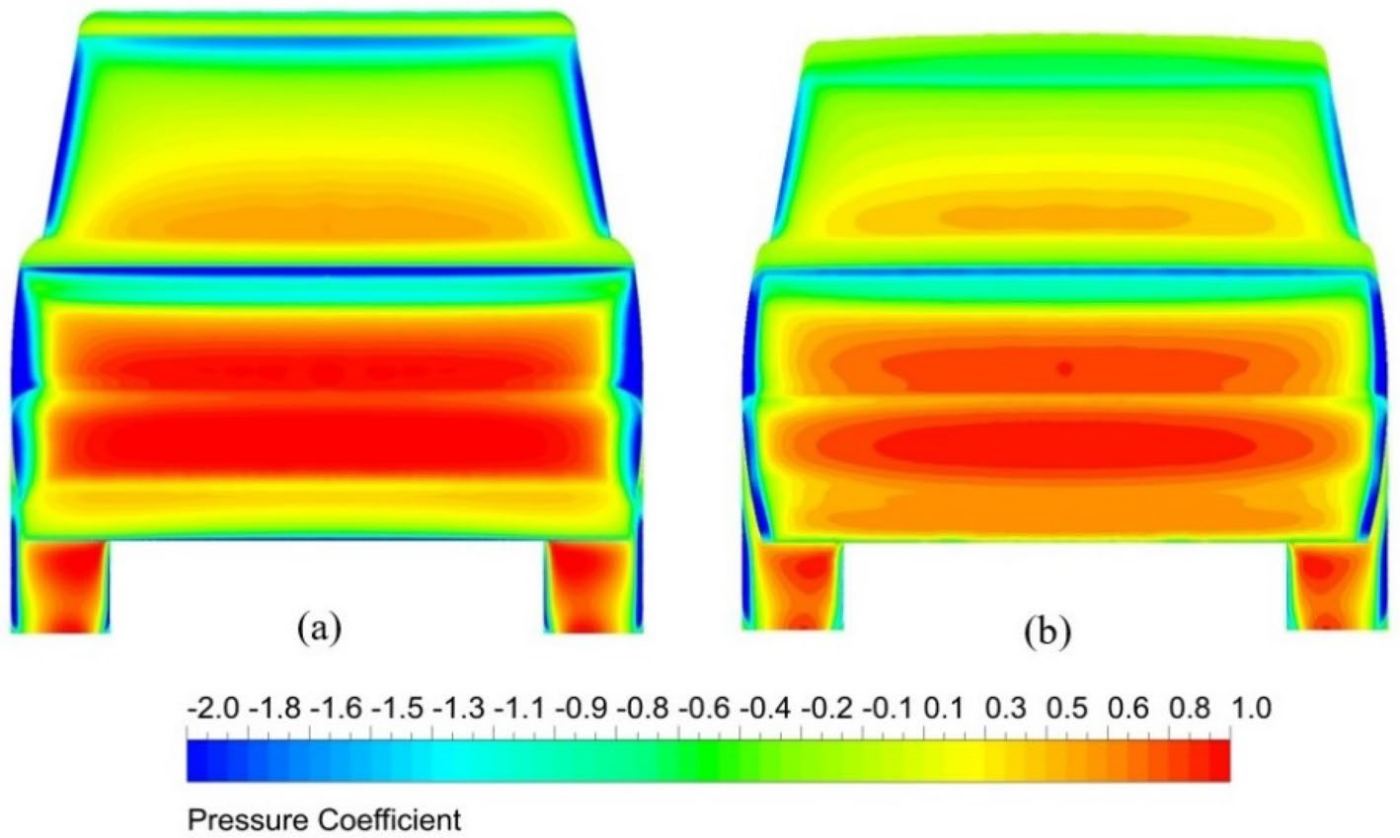
Front view of surface pressure distribution for two models of the LRD4: (**a**) the benchmark and (**b**) the modified model with diffuser and spare tire.

**Figure 16:**
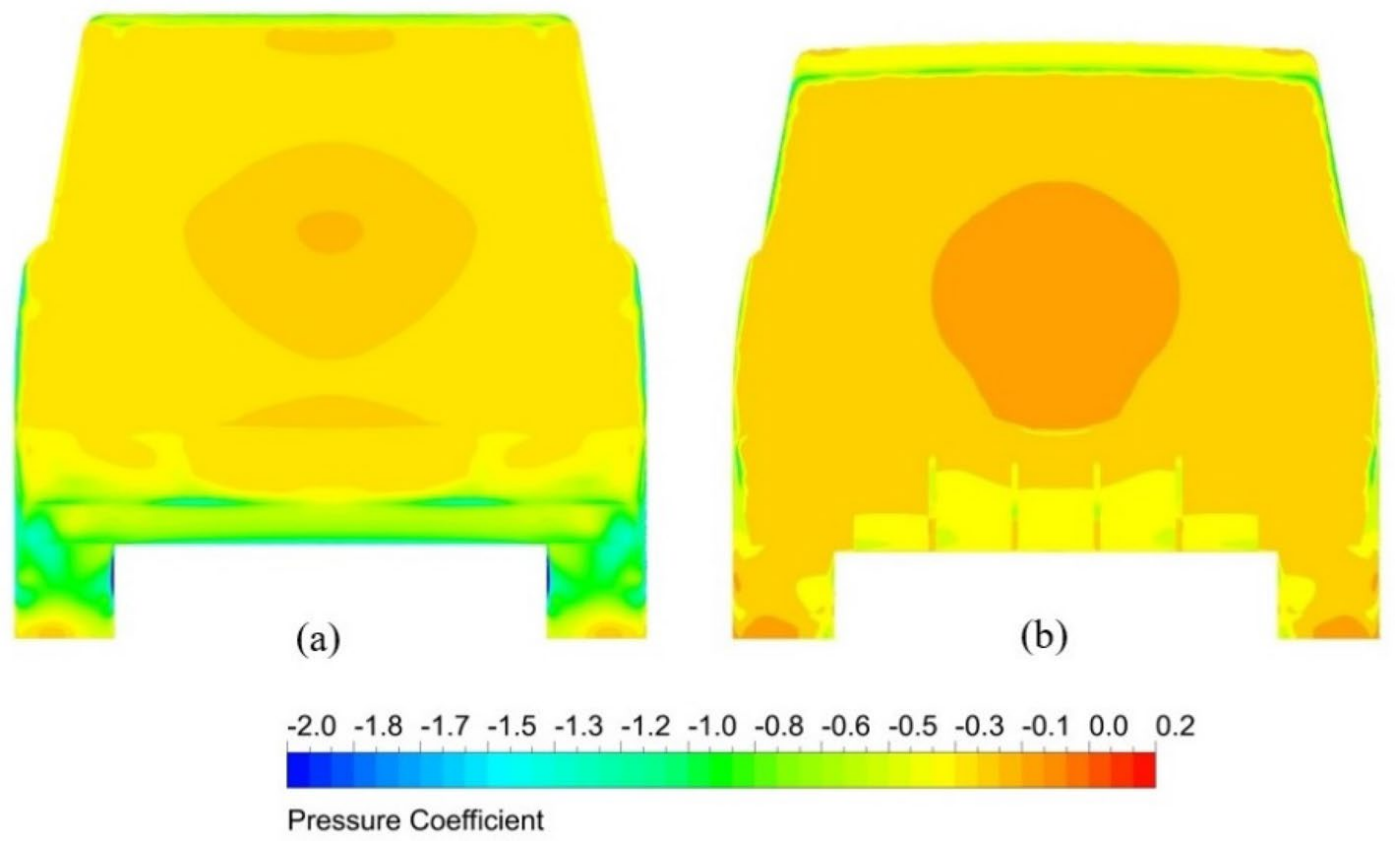
Back view of surface pressure distribution for two models of the LRD4: (**a**) the benchmark and (**b**) the modified model with diffuser and spare tire.

Representation of pressure distributions on the vehicle surface is done using the pressure coefficient ($C_{p}$). The scale of $C_{p}$ in the front of the vehicle is larger than in the rear region of the vehicle. A range of $C_{p}$ between − 2 and 1 are used in Fig. 15 for the front view of surface pressure distribution. While for the back view in Fig. 16, a range of $C_{p}$ between − 2 and 0.2 is used. Figure 15 shows the benchmark’s pressure distribution on the body surfaces and the optimal combined model. The red color indicates high pressure at the front of this SUV, especially in the middle of the front bumper, headlights, and grille. Hence the air resistance increases. However, modifying and adding some aerodynamic devices to the LRD4 reduces air resistance, as shown in Fig. 15b. Benchmark and optimal modified model have low pressure in A-pillars, front of the bonnet, front of the roof, and sidelights. Generally, the pressure coefficient on the external surfaces of the optimal modified LRD4 is more homogenous and smoother than the benchmark. The Discovery car's roof and front portion are curved, which helps minimize drag and increases positive lift force. Therefore, striking a balance between curved and flat surfaces is necessary. Aerodynamic tools and changes to the exterior design can be utilized to do this without reducing the vehicle's capacity.

Figure 16 illustrates the pressure distribution on the exterior surfaces of the LRD4 in the back view for the benchmark and optimal modified model. It is clear that most pressure variations of LRD4 occur in the back region of this model. Most pressures behind SUVs are negative, increasing air resistance. Two low-pressure regions at the rear part of the LRD4 are evident at the rear bumper and near the roof's trailing edge. Redesigning the LRD4 and adding a spare tire and diffuser can increase the pressure behind this SUV, as shown in Fig. 16b.

## Conclusion

The massive number of LRD4 configurations are investigated in the present study using the realizable $k{-}\varepsilon$ turbulence model to balance $C_{D}$ and $C_{L}$. Five different velocities for benchmark and optimal model of each modified model are investigated to study the effect of $Re$ on the $C_{D}$. Various configurations for each modification (redesigning the exterior shape of the LRD4, placing a spare tire on the back door of the LRD4, and adding a diffuser to the rear part of the LRD4 are investigated. In addition to the one combined modification of the LRD4 to achieve the minimum $C_{D}$. The number of cells within the global computational domain can affect the simulation results. Therefore, it should be tested to achieve an accurate simulation. The most important part regarding the drag coefficient is behind the car because the main vortices occur there. The curvature of the roof decreases drags while increasing lift. The increasing frontal area of the vehicle leads to an increase in the drag coefficient. The best $C_{L}$ is achieved by placing the spare tire on the back door (configuration 4) to increase the pressure above the rear part of this vehicle. On the other hand, visibility through the rear windshield is reduced. The vortices behind the LRD4 are irregular due to low pressure in this region. These vortices are dramatically reduced by adding a diffuser under the rear part of the LRD4. This aerodynamic device works to direct the airflow through the divergent passages. The drag coefficient of the Discovery benchmark is 0.4, while the modified exterior design is 0.361, which reduces about 9.75%. A diffuser can reduce *C*_*D*_ by approximately 10.25% (*C*_*D*_ = 0.359), and adding a spare tire to the back door can reduce *C*_*D*_ by 10.75% (*C*_*D*_ = 0.357). The best modifications in the current study in terms of $C_{D}$ (*C*_*D*_ = 0.352) and $C_{L}$ (*C*_*L*_ = 0.037) simultaneously are achieved by combining changes of redesigning the exterior shape, placing a spare tire on the back door, and adding a diffuser underbody. This configuration has reduced *C*_*D*_ by about 12%.

## Date availability

All data generated or analyzed during this study are included in this published article.
